# Recreational screen time and obesity risk in Korean children: a 3-year prospective cohort study

**DOI:** 10.1186/s12966-024-01660-0

**Published:** 2024-09-30

**Authors:** Hajin Jang, Yoonkyoung Cho, Hannah Oh

**Affiliations:** 1 Interdisciplinary Program in Precision Public Health, Department of Public Health Sciences, Graduate School of Korea University, 145 Anam-ro, Seongbuk-gu, Hana Science Building B. Room 358, Seoul, Republic of Korea; 2 School of Public Health, University of Pittsburgh, 130 De Soto St, Pittsburgh, PA USA; 3 Division of Health Policy and Management, College of Health Sciences, Korea University, 145 Anam-ro, Seongbuk-gu, Hana Science Building B. Room 358, Seoul, Republic of Korea

**Keywords:** Screen time, Screen device, Smartphone, Computer, Television, Internet, Media, Digital, Overweight, Children, Adolescent

## Abstract

**Background:**

Studies have shown that prolonged television watching increases obesity risk among children. However, few studies examined the associations with other types of screen time, such as computer and smartphone use, using a prospective cohort study design. Further, little is known about the specific non-screen time activity that may yield the most benefits when reallocating screen time to other activities.

**Methods:**

We conducted a prospective cohort analysis using 3-year follow-up data from the Korean Children and Youth Panel Survey 2018 (*n* = 2,023; 4th grade elementary students who were not obese at baseline). Average time spent watching television, using computer and smartphone, and other after-school activities were self-reported at baseline. Weight and height were also self-reported at baseline and follow-up surveys through 2021. We performed multivariable logistic regression models to estimate odds ratios (ORs) and 95% confidence intervals (CIs) for the associations between screen time and obesity incidence, adjusting for potential confounders. We also performed isotemporal substitution models to examine the associations of reallocating screen time to other non-screen time activities (physical activity, sleeping, hanging out with friends, reading, studying, and chatting with parents) in an equal time-exchange manner.

**Results:**

Longer combined screen time (≥ 240 vs. <120 m/d) was statistically significantly associated with an increased obesity risk (OR [95% CI] = 1.68 [1.03, 2.73]). The direction of associations with television watching (≥ 180 vs. <60 m/d: OR [95% CI] = 2.86 [1.58, 5.20]), computer use (≥ 120 vs. <60 m/d: 1.38 [0.52, 3.64]), and smartphone use (≥ 180 vs. <60 m/d: 1.42 [0.76, 2.65]) were all positive, although the association was most apparent and statistically significant for television watching only. The associations did not change after additional adjustment for other lifestyle factors, including physical activity, sleep, and breakfast skipping. In the isotemporal substitution models, reallocating 1-hour of screen time to reading (OR [95% CI] = 0.67 [0.48, 0.93]) was associated with a decreased obesity risk. Reallocating 1-hour of screen time to physical activity was only marginally significantly associated with obesity risk (0.79 [0.62, 1.01]).

**Conclusions:**

Our data suggest that more efforts should focus on reducing screen time and increasing time for other non-screen time activities, particularly reading, for obesity prevention in children.

**Supplementary Information:**

The online version contains supplementary material available at 10.1186/s12966-024-01660-0.

## Background

The prevalence of childhood obesity has increased worldwide over the past several decades [1]. Studies suggest that prolonged screen time, such as television watching, increases the risk of obesity among children [2–6]. In randomized controlled trials [4, 5], the school-based interventions that reduced television watching led to a decrease in body mass index (BMI) and other adiposity measures. During the past decade, other types of screen time, such as smartphone and computer use, also substantially increased, contributing to a large proportion of recreational time among children. Compared with television, smartphone and computer provide access to a wider range of contents (e.g., games, videos, social media) that may influence children’s food perception [7–13], dietary patterns [14–16], and other obesogenic behaviors (e.g., poor sleep pattern, physical inactivity) [17–22]. Smartphones are also more likely to be used for social purposes [23], such as sharing lifestyle and health information with peers via online platforms. While all types of screen time (e.g., television watching, smartphone use, computer use) are likely to displace physical activity and increase sedentary time, the types of content (e.g., games, videos, social media) and environmental setting (e.g., time of the day, location) to which children are frequently exposed during screen time may vary across different types of screen time. However, the associations of computer and smartphone use with obesity risk are not yet clear and little is known about whether the association varies by the type of screen time. Some cross-sectional studies reported positive associations of smartphone and computer use with the prevalence of childhood obesity [24–28] but reverse causation may have biased the associations in the cross-sectional studies. Therefore, prospective studies are required to accurately quantify the associations of multiple types of screen time, particularly smartphone and computer use, with obesity incidence.

Given the evidence suggesting negative health effects of prolonged screen time, many guidelines recommend reducing screen time in children. With the finite 24-hour a day, one-hour reduction in screen time should result in one-hour increment in other types of activity. However, little is known about the specific non-screen time activity (e.g., physical activity, sleeping, reading) that may replace screen time for most effective obesity prevention (i.e., substitution effect). Considering this issue, the isotemporal substitution models can statistically estimate the association with the specific activity being performed and the specific activity being displaced in an equal time-exchange manner [29, 30], allowing interpretations that are more relevant to public health recommendations.

In this study, we examined the prospective associations between multiple types of screen time (television watching, computer use, smartphone use) and obesity risk among the 4th grade Korean children, using the 3-year follow-up data. We also used the isotemporal substitution models to investigate the associations of reallocating screen time to other non-screen time activities. In the isotemporal substitution models, we included non-screen time activities that children of this age are commonly engaged during the 24-hour period (physical activity, sleeping, hanging out with friends, reading, studying, and chatting with parents) to identify the specific non-screen time activity that may yield the most benefit.

## Methods

### Study population

We conducted a prospective cohort analysis using 3-year follow-up data from the Korean Children and Youth Panel Survey 2018 (KCYPS 2018) 4th graders cohort, executed by the National Youth Policy Institute. The KCYPS 2018 is an ongoing cohort, including a nationally representative sample of 4th grade students in elementary school that was selected in 2018 using a multi-stage stratified sampling scheme according to geographical areas. The KCYPS 2018 participants have been followed up annually through 2021. The follow-up survey has been conducted every 2nd semester through 2021, with a follow-up rate of 87.3%. The information on behavioral and anthropometric factors is collected via in-person interviews during home visits. All participants and their parents (or guardians) provided informed consent and the study was approved by the Ethics Committee of the National Youth Policy Institute and Korea University (IRB-2022-0037).

Among 2,607 KCYPS 2018 participants who were in 4th grade in elementary school at baseline, we excluded participants who had missing information on weight and height at baseline (*n* = 13), those who were obese (BMI-for-age and -sex ≥ 95th percentile) at baseline (*n* = 269), and those who had missing follow-up information on weight and height through the end of follow up (*n* = 302). Similar baseline characteristics were observed among participants who were excluded due to having missing follow-up information (*n* = 302) compared with those who were included in the analysis (*n* = 2,023) (Supplementary Table 1). The final study population included 2,023 students (participant flowchart in Supplementary Fig. 1).

### Screen time and non-screen time activities

At baseline, participants were asked to report how they spent their time on an average day during the past semester. The following after-school activity items were asked: using smartphones for leisure, using computers for leisure, watching television for leisure, chatting with parents or caregivers, studying at private academies or tutoring, watching lectures online or on television, attending after-school classes, doing homework or studying alone, reading for leisure (excluding reading textbooks), exercising or performing physical activity after school, and hanging out with friends. Separately for weekdays and weekends, participants were asked to select one of the following categories: never, < 30 min, 30 min to < 1 h, 1 h to < 2 h, 2 h to < 3 h, 3 h to < 4 h, and ≥ 4 h. We then calculated the mean daily duration of each activity using the midpoint value of each category (the width of the open-ended extreme category was assumed to be the same as that of the adjacent category). Combined screen time was calculated as the sum of the time spent watching television, using computers, and using smartphones for leisure. Time spent studying at private academies or tutoring, watching lectures online or on television, attending after-school classes, and doing homework or studying were summed to represent combined study time. Separately for weekdays and weekends, participants were also asked to report the usual time going to bed at night and getting up in the morning. Based on the response, we calculated the average daily sleep duration. For the isotemporal substitution analysis, we estimated the average hours spent on each activity during the 24-hour day by assuming that the activities assessed in the study are an exhaustive list that participants performed during an average day. Because the duration of activities did not exactly add up to 24 h in some participants, we first calculated the proportion of time that each activity (using smartphones, using computers, watching television, performing physical activity, hanging out with friends, chatting with parents, studying, reading) contributed to participant’s summed duration of activities and then multiplied the proportion by participant’s total after-school hours (24 h minus sleeping and school hours). In the end, the activity times added up to 24 h within each participant, as indicated by the following Eq. (24 h = time using smartphones for leisure + time using computers for leisure + time watching televisions for leisure + time exercising or performing physical activity after school + time hanging out with friends + reading time + studying time + time chatting with parents + sleeping time + time at school).

### Obesity incidence

BMI was calculated as weight (kg) divided by height squared (m^2^), based on the weight and height data reported in baseline and follow-up surveys. For each participant, we identified the BMI percentile for age and sex based on the 2017 Korean National Growth Charts for children and adolescents [31]. Obesity was defined as having ≥ 95th BMI percentile for age and sex. Incident obesity was defined as obesity cases that were newly developed during the follow-up. Among 2,023 participants who did not have obesity at baseline, 133 participants developed incident obesity during the 3-year follow-up.

### Statistical analysis

We performed multivariable logistic regression models to estimate odds ratios (ORs) and 95% confidence intervals (CIs) of the associations between baseline screen time and 3-year obesity incidence. Because the exact date of obesity occurrence was not known, we used logistic regression models in our primary analysis. In the primary analysis, we used the baseline exposure variables to allow sufficient latency period between the exposure and outcome. In sensitivity analysis, we repeated the analysis using the Cox proportional hazards model with both baseline and time-varying exposures. All multivariable models included *a priori *selected potential confounders: sex, parental co-residence, parents’ highest education level, monthly household income, academic performance, and self-rated health (model 1). In model 2, we additionally adjusted for lifestyle factors, including breakfast skipping, physical activity, and sleep. Further adjustment for parents’ occupation and participants’ depressive symptom scores did not significantly change the results and thus these variables were excluded from the final models. To test for linear trends, we conducted the Wald test for continuous exposure variables (h/d). In secondary analysis, we examined the effect modification by sex because screen use and susceptibility to obesity are likely to be different between boys and girls. We tested for interaction using the Wald test for product terms. In the isotemporal substitution analysis [29, 30], we examined the association of reallocating screen time to other activity for the same amount of time. In the isotemporal substitution models, we included all activity time variables that contributed to participant’s 24-hour time composition, except for screen time variables (the activity being displaced), as follows: Logit (obesity) = β_0_ + (β_1_) time performing physical activity + (β_2_) sleeping time + (β_3_) time hanging out with friends + (β_4_) reading time + (β_5_) studying time + (β_6_) time chatting with parents + (β_7_) covariates. Time at school was not included in the model because it was constant for all participants in the same grade. The models leave out screen time variable, while holding other activity time variables constant. Given the finite 24-hour a day (i.e., isotemporal), a 1-h increase in specific activity (e.g., physical activity) in the model indicates a 1-h decrease in the activity variable that is left out from the model (screen time) (i.e., substitution). Each coefficient (β_1_ to β_6_) of activity time variable represents the association of 1-hour increment in the corresponding activity time in exchange of 1-hour decrement in screen time activity, while holding the other activity times constant.

All statistical tests were 2-sided with a 5% type I error rate. All analyses were conducted using SAS version 9.4 (SAS Institute).

## Results

### Study population

Table 1 presents the distribution of participant characteristics at baseline. The mean age at baseline was 10.2 years. The obesity incidence during the 3-year follow-up was 6.6%. Mean combined screen time was 2.5 h/d. Among different types of screen time, smartphone use (1.2 h/d) and television watching (1.1 h/d) contributed to the largest proportion of combined screen time. Compared to participants with < 120 min/d combined screen time, those with longer screen time were more likely to be boys, living with none or one parent and have lower parental education level, lower household income, poor academic performance, poor self-rated health, and shorter time spent reading, studying, and chatting with parents at baseline.

**Table 1:** Baseline characteristics of study participants in the KCYPS 2018

Characteristics	Combined screen time (min/d)			
	< 120 (*N* = 863)	120 to < 180 (*N* = 439)	180 to < 240 (*N* = 333)	≥ 240 (*N* = 388)
	***N*** **(%) or mean (SD)**			
**Age**,** years**	10.2 (0.3)	10.2 (0.3)	10.2 (0.3)	10.2 (0.3)
**Sex**				
Boys	404 (46.8)	214 (48.8)	169 (50.8)	203 (52.3)
Girls	459 (53.2)	225 (51.3)	164 (49.3)	185 (47.7)
**Parental co-residence**				
None or one parent	41 (4.8)	29 (6.6)	31 (9.3)	48 (12.4)
Both parents	822 (95.3)	410 (93.4)	302 (90.7)	340 (87.6)
**Parents’ highest education**				
High school or lower	108 (12.5)	81 (18.5)	78 (23.4)	102 (26.3)
College or higher	755 (87.5)	358 (81.6)	255 (76.6)	286 (73.7)
**Monthly household income**,** KRW**				
< 2,000,000	36 (4.2)	18 (4.1)	19 (5.7)	35 (9.0)
2,000,000 to < 4,000,000	190 (22.0)	139 (31.7)	93 (27.9)	119 (30.7)
4,000,000 to < 6,000,000	369 (42.8)	155 (35.3)	134 (40.2)	145 (37.4)
6,000,000 to < 8,000,000	145 (16.8)	74 (16.9)	59 (17.7)	58 (15.0)
≥ 8,000,000	123 (14.3)	53 (12.1)	28 (8.4)	31 (8.0)
**Academic performance**				
Poor or fair	217 (25.1)	128 (29.2)	104 (31.2)	146 (37.6)
Good	609 (70.6)	293 (66.7)	218 (65.5)	216 (55.7)
Missing	37 (4.3)	18 (4.1)	11 (3.3)	26 (6.7)
**Self-rated health**				
Very poor or poor	33 (3.8)	17 (3.9)	17 (5.1)	29 (7.5)
Good or very good	830 (96.2)	422 (96.1)	316 (94.9)	359 (92.5)
**Obesity incidence** ^**a**^	40 (4.6)	34 (7.7)	24 (7.2)	35 (9.0)
**Average duration of daily activities**,** h/d**				
Combined screen time	1.4 (0.9)	2.6 (0.8)	3.4 (1.0)	4.3 (1.4)
Television watching	0.7 (0.6)	1.1 (0.7)	1.3 (0.7)	1.7 (1.0)
Computer use	0.1 (0.3)	0.2 (0.4)	0.3 (0.6)	0.5 (0.8)
Smartphone use	0.6 (0.6)	1.2 (0.8)	1.7 (1.0)	2.1 (1.2)
Performing physical activity	1.2 (0.9)	1.1 (0.8)	1.1 (0.8)	1.0 (0.7)
Sleeping	9.4 (0.8)	9.3 (0.7)	9.3 (0.7)	9.2 (0.8)
Hanging out with friends	1.3 (1.0)	1.4 (0.9)	1.2 (0.8)	1.3 (0.9)
Reading	1.0 (0.8)	0.7 (0.6)	0.5 (0.5)	0.4 (0.4)
Studying	4.2 (1.5)	3.6 (1.3)	3.4 (1.2)	2.9 (1.3)
Chatting with parents	1.9 (1.2)	1.8 (1.1)	1.6 (1.0)	1.4 (0.9)

*Abbreviation* KCYPS, Korean Children and Youth Panel Survey; SE, standard error

^a^ Obesity was defined as body mass index for age and sex ≥ 95th percentile based on the 2017 Korean National Growth Charts

### Association between screen time and obesity

Table 2 presents the associations between screen time and obesity risk during the 3-year follow-up. Longer combined screen time (≥ 240 vs. <120 m/d) was statistically significantly associated with an increased obesity risk (OR [95% CI] = 1.68 [1.03, 2.73]; p-trend = 0.02; model 1). When we separately examined different types of screen time, television watching was significantly positively associated with an increased obesity risk (≥ 180 vs. <60 m/d: OR [95% CI] = 2.86 [1.58, 5.20]; p-trend = 0.04). For computer (≥ 120 vs. <60 m/d: OR [95% CI] = 1.38 [0.52, 3.64]; p-trend = 0.76) and smartphone use (≥ 180 vs. <60 m/d: OR [95% CI] = 1.42 [0.76, 2.65]; p-trend = 0.07), the direction of associations were suggestively positive but the associations were not statistically significant. Results did not change after additional adjustment for other lifestyle factors (breakfast skipping, physical activity, and sleep) in model 2.

**Table 2:** Multivariable-adjusted odds ratios (ORs) and 95% confidence intervals (CIs) for the associations between baseline screen time and obesity risk in a 3-year follow-up study, 2018–2021

Type of screen time	Cases / *N*	Model 1 OR (95% CI) ^a^	Model 2 OR (95% CI) ^b^
**Combined screen time (m/d)**			
< 120	40/863	1.00 (ref)	1.00 (ref)
120 to < 180	34/439	1.65 (1.02, 2.67)	1.65 (1.02, 2.67)
180 to < 240	24/333	1.43 (0.84, 2.44)	1.40 (0.82, 2.40)
≥ 240	35/388	1.68 (1.03, 2.73)	1.68 (1.02, 2.76)
p-trend ^c^		0.02	0.02
**Television watching (m/d)**			
< 60	74/1,215	1.00 (ref)	1.00 (ref)
60 to < 120	33/535	1.02 (0.66, 1.57)	1.03 (0.67, 1.58)
120 to < 180	9/179	0.76 (0.37, 1.55)	0.76 (0.37, 1.56)
≥ 180	17/94	2.86 (1.58, 5.20)	2.98 (1.63, 5.44)
p-trend ^c^		0.04	0.03
**Computer use (m/d)**			
< 60	122/1,865	1.00 (ref)	1.00 (ref)
60 to < 120	6/107	0.68 (0.29, 1.60)	0.66 (0.28, 1.57)
≥ 120	5/51	1.38 (0.52, 3.64)	1.34 (0.50, 3.58)
p-trend ^c^		0.76	0.83
**Smartphone use (m/d)**			
< 60	63/1,117	1.00 (ref)	1.00 (ref)
60 to < 120	37/518	1.18 (0.76, 1.81)	1.17 (0.76, 1.80)
120 to < 180	18/231	1.22 (0.70, 2.14)	1.23 (0.70, 2.16)
≥ 180	15/157	1.42 (0.76, 2.65)	1.36 (0.72, 2.57)
p-trend ^c^		0.07	0.09

^a^ Model 1 included sex (boy/girl), parental co-residence (none or one parent/both parents), parents’ highest education level (high school or lower/college or higher), monthly household income (< 200/200 to <400/400 to <600/600 to <800/≥800, unit: 10,000 KRW), academic performance (poor or fair/good/unknown), and self-rated health (very poor or poor/good or very good)

^b^ Model 2 included all variables in model 1 plus breakfast skipping (0–4/≥5 d/w), average duration of physical activity (h/d, continuous), and average duration of sleep (h/d, continuous)

^c^ P-trend was estimated using the Wald test for continuous exposure variable (h/d)

### Isotemporal substitution analysis

**Fig. 1:**
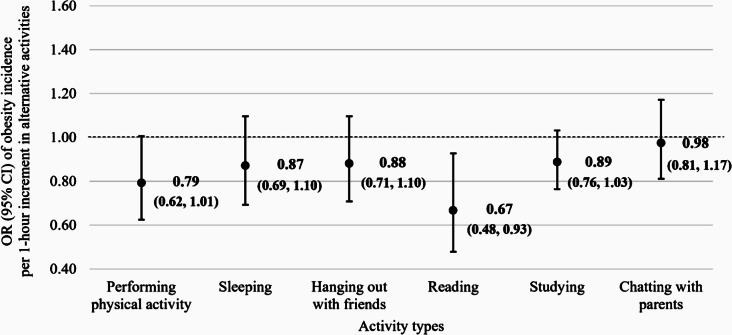
Multivariable-adjusted odds ratios (ORs) and 95% confidence intervals (CIs) of obesity incidence associated with reallocating 1-hour of combined screen time to non-screen time activities in a 3-year follow-up study, 2018–2021. The graph shows the multivariable-adjusted ORs and 95% CIs of obesity incidence associated with increasing 1-hour of non-screen time activities (performing physical activity, sleeping, hanging out with friends, reading, studying, chatting with parents) while decreasing 1-hour of combined screen time. Multivariable-adjusted models included sex (boy, girl), parental co-residence (none or one parent, both parents), parents’ highest education (high school or lower, college or higher), monthly household income (< 2,000,000; 2000,000 to < 4,000,000; 4,000,000 to < 6,000,000; 6,000,000 to < 8,000,000; ≥8,000,000 KRW), academic performance (poor or fair, good, missing), and self-rated health (very poor or poor, good or very good), and all non-screen time activities (performing physical activity, sleeping, hanging out with friends, reading, studying, chatting with parents)

Figure 1 shows the results from the isotemporal substitution analysis. In the isotemporal substitution models, substituting an 1-h/d of reading for an 1-h/d of screen time was statistically significantly associated with a lower obesity risk (OR [95% CI] = 0.67 [0.48, 0.93]). The direction of association of substituting an 1-h/d of physical activity for an 1-h/d of screen time was suggestively positive (OR [95% CI] = 0.79 [0.62, 1.01]) but the association was not statistically significant. Other non-screen time activities, including sleeping, hanging out with friends, studying, and chatting with parents, were not statistically significantly associated.

When stratified by sex, similar results were observed in boys and girls (Supplementary Table 2). Similar results were also observed when Cox proportional hazards models, instead of logistic regression models, were performed with baseline (Supplementary Table 3, Supplementary Fig. 2) and time-varying exposures (Supplementary Table 4).

## Discussion

In this prospective analysis, we observed that longer combined screen time was associated with an increased risk of obesity among Korean children. When different types of screen time were evaluated, the positive association was statistically significant with television watching only. In the isotemporal substitution analyses, reallocating screen time to reading was associated with a reduced obesity risk. Our data suggest that reducing screen time and increasing time for reading may bring the most benefit in obesity prevention.

Our findings of positive association between screen time and obesity risk are consistent with those from previous studies [2–6]. Many observational [2, 3, 6] and experimental studies [4, 5] support that longer television watching increases the risk of obesity in children and adolescents. Some studies also demonstrated that the increased obesity risk may last through adulthood [32, 33], further emphasizing the importance of reducing screen time at young ages. Our findings are also consistent with the current guidelines that recommend less than 2 h/d of screen time in children [34, 35], as we observed an increased obesity risk at ≥ 2 h/d of combined screen time. While most studies of modern screen time, such as computer and smartphone use, have been cross-sectional [24–28], we examined the associations of different types of screen time with obesity incidence using a 3-year longitudinal study. In our study, the direction of association was suggestively positive for all types of screen time, including computer and smartphone use, although the positive association was statistically significant and most apparent for television watching only. Statistically nonsignificant associations with computer and smartphone use may be due to the limited sample size and limited range of exposure for each screen type use. Our data suggest that television watching may have the most negative influence on obesity risk among children. However, given the wide range of contents that are accessible on smartphones and computers, further studies are needed to investigate whether the associations vary by the specific contents and information that children access using these screen devices.

There are several potential mechanisms that may explain the link between screen time and obesity risk among children. Screen time may increase the opportunity for snacking, resulting in increased consumption of high-calorie, ultra-processed foods (e.g., sugar-sweetened beverages, chips/crackers, instant foods, ready-meals) [14, 15] that are associated with elevated risks of obesity [36, 37] and other chronic disease [38, 39]. Eating during screen time may also promote “mindless eating” (eating without acknowledging the quantity and quality of foods), leading to overeating and weight gain. In addition, during screen time, children are more likely to be exposed to advertisements and marketing of unhealthy foods (e.g., fast foods, sugar-sweetened beverages) [40, 41], which may influence their food preferences and food choices [14, 15]. Using blue light-emitting screen devices, such as televisions, computers, and smartphones, before bedtime may also delay sleep onset and disrupt sleep quality by suppressing melatonin secretion [42, 43]. Insufficient sleep may alter appetite and metabolism and thereby increase weight gain [44]. Longer screen time may also indicate shorter time available for healthier activities, such as physical activity, that require higher energy expenditure. In our study, we observed that the positive association between screen time and obesity risk was persistent after additional adjustment for physical activity, sleep, and breakfast skipping, suggesting that the associations may be independent of these factors. Further studies are needed to examine the potential mechanisms that may link screen time and obesity risk in children.

In our isotemporal substitution analysis, we also observed that reallocating screen time to reading was associated with a reduced obesity risk. Consistent with our findings, a previous study of Chinese 1st to 3rd grade elementary students reported that reallocating screen time to other sedentary activities, including reading, was associated with lower BMI [45]. Compared with screen device use, reading is likely to involve more active brain engagement and higher energy expenditure [46]. During reading, children can also avoid the exposure to advertisements and marketing of unhealthy foods. While reading, children are also likely to obtain more accurate health information compared with screen time, as many digital contents present misinformation [47]. Reading may also be associated with other healthier lifestyle choices (e.g., healthier food choice). Further studies are needed to explore the mechanisms that may link the association between reading and obesity risk in children. In our study, we also observed that reallocating screen time to physical activity was suggestively associated with a lower obesity risk, although the association was not statistically significant. Physical activity can increase total energy expenditure and prevent weight gain. Physical activity also provides additional health benefits such as improving cardiometabolic profile [48] and reducing depression and anxiety [49]. Children are less likely to consume extra calories when performing physical activity and reading compared with when watching television or playing games on smartphones, leading to lower total caloric intake.

We acknowledge that this study has several limitations. We used self-reported data of screen time and anthropometric measures and thus our results may include measurement errors. Our data were also collected via face-to-face interview of students. For this reason, screen time and obesity may have been under-reported due to social desirability bias. Further, although children may simultaneously use multiple screens (e.g., using a smartphone while watching television), we were not able to examine the association with simultaneous use of multiple screen types due to lack of data. Lastly, our study included 4th grade Korean students and thus our results may not be generalizable to populations of different age and ethnicity who have different susceptibility to obesity.

Despite the limitations, this study has important strengths. While most studies of smartphone and computer use were cross-sectional, this study examined the associations with obesity risk using a prospective cohort study with a 3-year follow-up. We also included 3 different types of screen time and compared the associations across different types. Further, our study provided additional insights into the specific activity that may be most effective in obesity prevention when reallocating screen time.

## Conclusions

In our 3-year follow-up study, we observed that longer combined screen time was positively associated with obesity risk among Korean children. Reallocating screen time to physical activity or reading was associated with decreased obesity risk. For obesity prevention, efforts should focus on reducing all types of screen time while increasing time for physical activity or reading.

## Electronic supplementary material

Below is the link to the electronic supplementary material.

Supplementary Material 1

Supplementary Material 2

## Data Availability

Korean Children and Youth Panel Survey 2018 data, collected by National Youth Policy Institute (NYPI), were used and can be accessed through NYPI Youth and Children Data Archive (https://www.nypi.re.kr/archive/mps).

## Abbreviations

BMI: Body mass index
CI: Confidence interval
KCYPS: Korean Children and Youth Panel Survey
OR: Odds ratio
