# Supplementary material for: Recreational screen time and obesity risk in Korean children: a 3-year prospective cohort study
Source: Int J Behav Nutr Phys Act. 2024 Sep 30;21:112. doi: 10.1186/s12966-024-01660-0 (PMC11440942; doi:10.1186/s12966-024-01660-0)
Supplement: Supplementary file 2 — Supplementary Material 2 [file 12966_2024_1660_MOESM2_ESM.docx]

**Supplementary Figure 1. Flow chart of study participants**

**2,607** 4^th^ grade students from the Korean Children and Youth Panel Survey 2018 (KCYPS 2018)

Excluded **13** students with missing information on weight and height at baseline

**2,594** students with weight and height reported at baseline

Excluded **269** students who were obese (BMI-for-age and -sex ≥95th percentile) at baseline

**2,325** students who were not obese (BMI-for-age and -sex ≥95th percentile) at baseline

Excluded **302** students who had missing follow-up information on weight and height through the end of follow-up

**2,023** final study population

**Supplementary Figure 2. Multivariable-adjusted hazard ratios (HRs) and 95% confidence intervals (CIs) of obesity risk associated with reallocating 1-hour of combined screen time to non-screen time activities in a 3-year follow-up study, 2018-2021^a^**

**
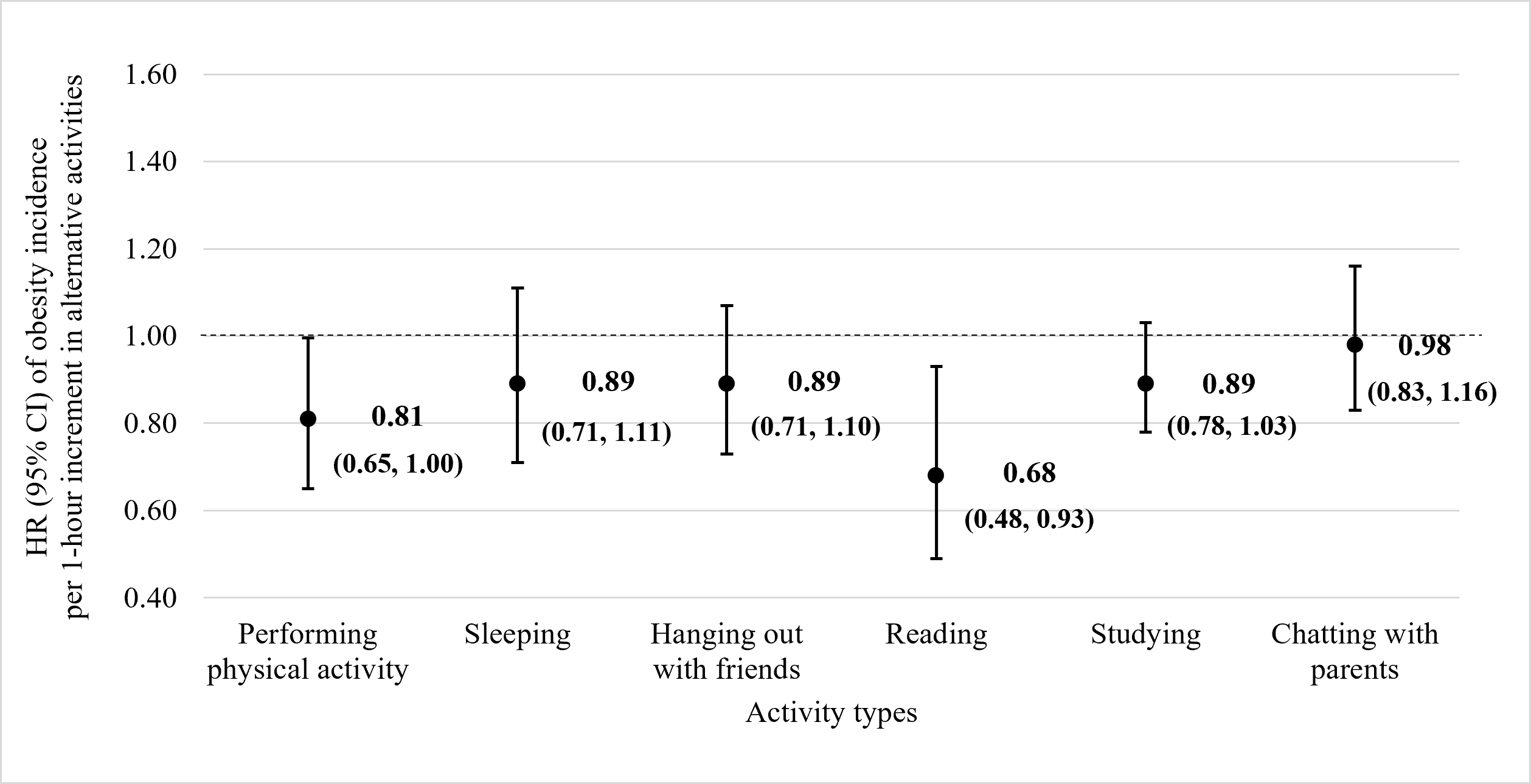
**

^a^ The graph shows the multivariable-adjusted HRs and 95% CIs of obesity risk associated with increasing 1-hour of non-screen time activities (performing physical activity, sleeping, hanging out with friends, reading, studying, chatting with parents) while decreasing 1-hour of combined screen time. Multivariable model adjusted for sex (boy, girl), parental co-residence (none or one parent, both parents), parents’ highest education (high school or lower, college or higher), monthly household income (<2,000,000; 2000,000 to <4,000,000; 4,000,000 to <6,000,000; 6,000,000 to <8,000,000; ≥8,000,000 KRW), academic performance (poor or fair, good, missing), and self-rated health (very poor or poor, good or very good), and all non-screen time activities (performing physical activity, sleeping, hanging out with friends, reading, studying, chatting with parents).

**Supplementary Table 1. Comparison of characteristics between included participants and participants lost to follow-up**

| **Characteristics** | **Participants included in the analysis**  **(N=2,023)** | **Participants excluded due to having missing follow-up information**  **(N-302)** | ***P*-value ^a^** |
| --- | --- | --- | --- |
|  | **N (%) or mean (SD)** | |  |
| **Age (years)** | 10.2 (0.3) | 10.2 (0.3) | 0.35 |
| **Sex** |  |  |  |
| Boys | 990 (48.9) | 149 (49.3) | 0.90 |
| Girls | 1,033 (51.1) | 153 (50.7) |  |
| **Parental co-residence** |  |  | 0.12 |
| None or one parent | 149 (7.4) | 30 (9.9) |  |
| Both parents | 1,874 (92.6) | 272 (90.1) |  |
| **Parents’ highest education** |  |  | 0.41 |
| High school or lower | 369 (18.2) | 61 (20.2) |  |
| College or higher | 1,654 (81.8) | 241 (79.8) |  |
| **Monthly household income (KRW)** |  |  | 0.75 |
| <2,000,000 | 108 (5.3) | 17 (5.6) |  |
| 2,000,000 to <4,000,000 | 541 (26.7) | 76 (25.2) |  |
| 4,000,000 to <6,000,000 | 803 (39.7) | 127 (42.1) |  |
| 6,000,000 to <8,000,000 | 336 (16.6) | 43 (14.2) |  |
| ≥8,000,000 | 235 (11.6) | 39 (12.9) |  |
| **Academic performance** |  |  | 0.39 |
| Poor or fair | 595 (29.4) | 84 (27.8) |  |
| Good | 1,336 (66.0) | 199 (65.9) |  |
| Missing | 92 (4.6) | 19 (6.3) |  |
| **Self-rated health** |  |  | 0.27 |
| Very poor or poor | 96 (4.8) | 10 (3.3) |  |
| Good or very good | 1,927 (95.3) | 292 (96.7) |  |
| **Average duration of daily activities (h/d)** |  |  |  |
| Combined screen time | 2.5 (1.5) | 2.5 (1.6) | 0.65 |
| Television watching | 1.1 (0.8) | 1.0 (0.8) | 0.70 |
| Computer use | 0.3 (0.5) | 0.3 (0.5) | 0.90 |
| Smartphone use | 1.2 (1.0) | 1.2 (1.6) | 0.69 |
| Performing physical activity | 1.1 (0.8) | 1.1 (0.8) | 0.80 |
| Sleeping | 9.3 (0.8) | 9.3 (0.8) | 0.54 |
| Hanging out with friends | 1.3 (0.9) | 1.4 (0.9) | 0.26 |
| Reading | 0.7 (0.7) | 0.6 (0.6) | <0.01 |
| Studying | 3.7 (1.5) | 3.7 (1.4) | 0.57 |
| Chatting with parents | 1.7 (1.1) | 1.7 (1.1) | 0.99 |

Abbreviation: KCYPS, Korean Children and Youth Panel Survey; SE, standard error.

^a^ P-value was estimated using the chi-square test for categorical variables and t-test for continuous variables

**Supplementary Table 2. Multivariable-adjusted odds ratios (ORs) and 95% confidence intervals (CIs) for the associations between baseline screen time and obesity risk in a 3-year follow-up study, stratified by sex**

|  | **Boys (N=990)** | | **Girls (N=1,033)** | | **p-int ^b^** |
| --- | --- | --- | --- | --- | --- |
| **Types of screen time** | **Cases / N** | **OR (95% CI) ^a^** | **Cases / N** | **OR (95% CI) ^a^** |  |
| **Combined screen time (m/d)** |  |  |  |  | 0.30 |
| <120 | 29/404 | 1.00 (ref) | 11/459 | 1.00 (ref) |  |
| 120 to <180 | 25/214 | 1.63 (0.92, 2.89) | 9/225 | 1.65 (0.67, 4.08) |  |
| 180 to <240 | 16/169 | 1.21 (0.63, 2.32) | 8/164 | 1.96 (0.76, 5.02) |  |
| **≥**240 | 23/203 | 1.36 (0.74, 2.32) | 12/185 | 2.65 (1.12, 6.25) |  |
| p-trend ^c^ |  | 0.21 |  | 0.01 |  |
| **Television watching (m/d) ^e^** |  |  |  |  | 0.16 |
| <60 | 54/595 | 1.00 (ref) | 20/620 | 1.00 (ref) |  |
| 60 to <120 | 23/247 | 1.03 (0.61, 1.74) | 10/288 | 1.03 (0.47, 2.25) |  |
| 120 to <180 | 5/94 | 0.53 (0.20, 1.37) | 4/85 | 1.52 (0.50, 4.61) |  |
| **≥**180 | 11/54 | 2.26 (1.08, 4.74) | 6/40 | 5.11 (1.86, 14.00) |  |
| p-trend ^c^ |  | 0.39 |  | 0.01 |  |
| **Computer use (m/d) ^e^** |  |  |  |  | 0.96 |
| <60 | 84/890 | 1.00 (ref) | 38/975 | 1.00 (ref) |  |
| 60 to <120 | 6/69 | 0.85 (0.35, 2.04) | 0/38 | Not estimated |  |
| **≥**120 | 3/31 | 1.01 (0.29, 3.49) | 2/20 | 3.12 (0.66, 14.70) |  |
| p-trend ^c^ |  | 0.74 |  | 0.90 |  |
| **Smartphone use (m/d) ^e^** |  |  |  |  |  |
| **<**60 | 45/545 | 1.00 (ref) | 18/572 | 1.00 (ref) | 0.64 |
| 60 to <120 | 25/255 | 1.08 (0.64, 1.83) | 12/263 | 1.41 (0.66, 3.01) |  |
| 120 to <180 | 15/115 | 1.43 (0.75, 2.73) | 3/116 | 0.75 (0.21, 2.66) |  |
| **≥**180 | 8/75 | 0.97 (0.42, 2.24) | 7/82 | 2.57 (0.98, 6.72) |  |
| p-trend ^c^ |  | 0.28 |  | 0.10 |  |

^a^ Adjusted for parental co-residence (none or one parent/both parents), parents’ highest education level (high school or lower/college or higher), monthly household income (<200/200-<400/400-<600/600-<800/≥800, unit: 10000 won), academic performance (poor or fair/good/unknown), and self-rated health (Very poor or poor/good or very good).

^b^ P-interaction was estimated using the Wald test for product term between sex and screen time variables

^c^ P-trend was estimated using the Wald test for continuous exposure variables (h/d)

**Supplementary Table 3. Multivariable-adjusted hazard rations (HRs) and 95% confidence intervals (CIs) for the associations between baseline screen time and obesity risk in a 3-year follow-up study, 2018-2021**

| **Types of screen time** | **Cases / Person-years** | **Model 1**  **HR (95% CI) ^b^** | **Model 2**  **HR (95% CI) ^c^** |
| --- | --- | --- | --- |
| **Combined screen time (m/d)** |  |  |  |
| <120 | 40/2552 | 1.00 (ref) | 1.00 (ref) |
| 120 to <180 | 34/1276 | 1.62 (1.03, 2.55) | 1.62 (1.02, 2.55) |
| 180 to <240 | 24/975 | 1.41 (0.86, 2.32) | 1.38 (0.84, 2.29) |
| **≥**240 | 35/1127 | 1.65 (1.06, 2.58) | 1.65 (1.04, 2.61) |
| p-trend ^d^ |  | 0.01 | 0.01 |
| **Television watching (m/d)** |  |  |  |
| <60 | 74/3575 | 1.00 (ref) | 1.00 (ref) |
| 60 to <120 | 33/1564 | 1.03 (0.69, 1.54) | 1.03 (0.69, 1.55) |
| 120 to <180 | 9/528 | 0.76 (0.38, 1.52) | 0.76 (0.38, 1.52) |
| **≥**180 | 17/263 | 2.64 (1.59, 4.38) | 2.74 (1.64, 4.58) |
| p-trend ^d^ |  | 0.03 | 0.03 |
| **Computer use (m/d)** |  |  |  |
| <60 | 122/5469 | 1.00 (ref) | 1.00 (ref) |
| 60 to <120 | 6/310 | 0.72 (0.32, 1.64) | 0.71 (0.31, 1.61) |
| **≥**120 | 5/151 | 1.30 (0.54, 3.13) | 1.26 (0.51, 3.12) |
| p-trend ^d^ |  | 0.75 | 0.85 |
| **Smartphone use (m/d)** |  |  |  |
| **<**60 | 63/3287 | 1.00 (ref) | 1.00 (ref) |
| 60 to <120 | 37/1517 | 1.16 (0.77, 1.74) | 1.15 (0.77, 1.73) |
| 120 to <180 | 18/668 | 1.25 (0.76, 2.08) | 1.27 (0.76, 2.11) |
| **≥**180 | 15/458 | 1.38 (0.78, 2.44) | 1.32 (0.73, 2.39) |
| p-trend ^d^ |  | 0.05 | 0.07 |

^b^ Model 1 included sex (boy/girl), parental co-residence (none or one parent/both parents), parents’ highest education level (high school or lower/college or higher), monthly household income (<200/200-<400/400-<600/600-<800/≥800, unit: 10000 won), academic performance (poor or fair/good/unknown), and self-rated health (Very poor or poor/good or very good).

^c^ Model 2 included all variables in model 1 plus breakfast skipping (0-4/≥5 d/w), average duration of physical activity (h/d, continuous), and average duration of sleep (h/d, continuous)

^d^ P-trend was estimated using the Wald test for continuous exposure variable (h/d)

**Supplementary Table 4. Multivariable-adjusted hazards ratios (HRs) and 95% confidence intervals (CIs) for the associations between screen time and obesity risk in a 3-year follow-up study, with updated time-varying exposure (screen time) during the follow-up**

| **Types of screen time** | **Cases / Person-years** | **Model 1**  **HR (95% CI) ^a^** | **Model 2**  **HR (95% CI) ^b^** |
| --- | --- | --- | --- |
| **Combined screen time (m/d)** |  |  |  |
| <120 | 51/3628 | 1.00 (ref) | 1.00 (ref) |
| 120 to <180 | 56/3122 | 1.81 (1.10, 3.00) | 1.80 (1.09, 2.98) |
| 180 to <240 | 41/2797 | 1.49 (0.87, 2.56) | 1.47 (0.86, 2.53) |
| **≥**240 | 59/3746 | 1.45 (0.88, 2.39) | 1.43 (0.86, 2.37) |
| p-trend ^c^ |  | 0.30 | 0.34 |
| **Television watching (m/d)** |  |  |  |
| <60 | 99/5639 | 1.00 (ref) | 1.00 (ref) |
| 60 to <120 | 59/3626 | 1.09 (0.73, 1.63) | 1.09 (0.73, 1.62) |
| 120 to <180 | 23/1521 | 1.18 (0.68, 2.07) | 1.19 (0.68, 2.07) |
| **≥**180 | 21/594 | 1.61 (0.80, 3.24) | 1.62 (0.81, 3.25) |
| p-trend ^d^ |  | 0.20 | 0.20 |
| **Computer use (m/d)** |  |  |  |
| <60 | 129/6123 | 1.00 (ref) | 1.00 (ref) |
| 60 to <120 | 20/1751 | 1.08 (0.64, 1.83) | 1.07 (0.64, 1.81) |
| **≥**120 | 16/946 | 0.98 (0.49, 1.96) | 0.98 (0.49, 1.97) |
| p-trend ^d^ |  | 0.93 | 0.94 |
| **Smartphone use (m/d)** |  |  |  |
| **<**60 | 74/4597 | 1.00 (ref) | 1.00 (ref) |
| 60 to <120 | 60/3915 | 1.09 (0.72, 1.65) | 1.08 (0.71, 1.64) |
| 120 to <180 | 32/2484 | 0.87 (0.51, 1.49) | 0.87 (0.51, 1.48) |
| **≥**180 | 29/1654 | 1.41 (0.83, 2.38) | 1.36 (0.80, 2.32) |
| p-trend ^d^ |  | 0.45 | 0.51 |

^a^ Model 1 included sex (boy/girl), parental co-residence (none or one parent/both parents), parents’ highest education level (high school or lower/college or higher), monthly household income (<200/200-<400/400-<600/600-<800/≥800, unit: 10000 won), academic performance (poor or fair/good/unknown), and self-rated health (Very poor or poor/good or very good).

^b^ Model 2 included all variables in model 1 plus breakfast skipping (0-4/≥5 d/w), average duration of physical activity (h/d, continuous), and average duration of sleep (h/d, continuous)

^c^ P-trend was estimated using the Wald test for continuous ordinal score (0-3)
